# Environmental Factors Drive the Biogeographic Pattern of *Hippophae rhamnoides* Root Endophytic Fungal Diversity in the Arid Regions of Northwest China

**DOI:** 10.3390/jof10100679

**Published:** 2024-09-29

**Authors:** Siyu Guo, Guisheng Ye, Wenjie Liu, Ruoqi Liu, Zhehao Liu, Yuhua Ma

**Affiliations:** College of Agriculture and Animal Husbandry, Qinghai University, Xining 810016, China; siyu_guo1005@outlook.com (S.G.); 2007990011@qhu.edu.cn (G.Y.); asd222333666@outlook.com (W.L.); ruoqi1002@outlook.com (R.L.); liuzhehao96@outlook.com (Z.L.)

**Keywords:** *Hippophae rhamnoides* subsp. *sinensis* rousi, biogeographic pattern, altitude, FUNGuild

## Abstract

*Hippophae rhamnoides* subsp. *sinensis* Rousi (Abbrev. *H. rhamnoides*) stands as a vital botanical asset in ameliorating the ecological landscape of the arid regions in Northwest China, where its rhizospheric microorganisms serve as linchpins in its growth and developmental dynamics. This study aimed to explore the community structure characteristics and origin differences of root endophytic fungi in *H. rhamnoides*. Samples were collected from 25 areas where *H. rhamnoides* is naturally distributed along an altitude gradient in the northwest region. Then, endophytic fungi from different regions were analyzed by using high-throughput sequencing technology to compare the structural characteristics of endophytic fungi and examine their association with environmental factors. FUNGuild was employed to analyze the community structure and functions of endophytic fungi, and the results showed that each region had its own dominant endophytic fungal flora, demonstrating the differences in origin of endophytic fungi, and the specific endophytic flora acquired from the original soil in the growing season of *H. rhamnoides* will help us construct the microecological community structure. Furthermore, the study identified and assessed the diversity of fungi, elucidating the species structure and highlighting dominant species. The RDA analysis revealed that available phosphorus (AP), available potassium (AK), and total nitrogen (TN) exhibit significant correlations with the composition and diversity of root-associated fungi. In conclusion, the fungal community structure is similar within the same region, while significant differences exist in the taxonomic structure and biodiversity among different regions. These findings shed light on the intricate interplay and mechanisms governing the ecological restoration of *H. rhamnoides*, offering a valuable framework for advancing green ecology initiatives and harnessing the potential of root-associated microorganisms in this species.

## 1. Introduction

Plant roots play a crucial role in ecological processes, which are widely perceived as contributing to plant health, growth, and biogeochemical cycles [1,2]. Endophytic fungi refers to fungi that live in plant tissues at specific or all stages of their life cycle but do not cause obvious harm to the plant [3,4]. Fungi in the rhizosphere play a crucial role in how plants cope with abiotic stresses caused by pathogenic bacteria [5,6,7] by improving the molecular exchange between soil microorganisms and root exudates, ultimately supporting healthy plant growth and triggering systemic resistance [8]. Root–microbe symbiotic relationships are key components of soil ecosystems [9], in which soil serves as the primary reservoir for numerous plant endophytes [10], and compounds released by plant roots have the ability to attract particular populations in the soil to the rhizosphere, leading to the formation of a rhizosphere microbial community closely intertwined with plants [11,12], so that the interactions among plants, microbes, and soil create a distinct soil ecosystem, offering a specialized growth environment conducive to plant development [13].

Previous studies have indicated that factors such as altitude, climate, soil variables, and vegetation characteristics significantly influence the composition and diversity of root endophytic fungal communities [14,15,16,17,18]. For instance, altitude can impact the diversity of root endophytic fungal communities, with noticeable differences along altitude gradients. Other factors, such as soil properties, climate, and vegetation, also affect the composition of fungi in plant rhizomes. Overall, there are varying degrees of correlation between geographical and climatic factors and the diversity of active ingredients in plants and fungi found within plant rhizomes. The environment plays a crucial role in determining the composition of fungi on a continental scale [19,20,21,22]. Root fungi exhibit similarities to soil microorganisms and respond quickly to soil microenvironments. Additionally, the fungi in plant rhizomes are not only influenced by the environment itself but also by plant types and genotypes [23]. As a more specialized environment, rhizomes exert a stronger selective pressure on fungal communities. Root-associated fungi facilitate nutrient absorption for plants, provide defense against pathogenic microorganisms, and regulate plant growth and development in response to the surrounding soil microenvironment [12,24].

*Hippophae rhamnoides*, a plant belonging to the genus *Hippophae* in the family Elaeagnaceae, is a pioneer tree species customarily used for afforestation in arid regions [25]. Its native drought-tolerant nature has made it particularly popular in Northwest China and has garnered significant attention from scientific researchers in recent years [26,27,28]. While some studies have investigated the rhizosphere soil microorganisms of *H. rhamnoides* [29,30,31], research on the endophytic fungi within its roots remains limited. Current studies primarily focus on the edible [32] and ecological [33,34] values of *H. rhamnoides*. The microecological structure of its rhizomes is largely unexplored, and the correlation with environmental factors has yet to be thoroughly investigated. This study endeavors to conduct an analysis and comparison of natural populations of *H. rhamnoide* across 25 research sites situated in the arid regions of Northwest China, employing advanced high-throughput sequencing technology (ITS) in conjunction with the FUNGuild database. This study will use high-throughput sequencing and bioinformatics to analyze root endophytic fungi, focusing on their community structure, functional characteristics, network relationships, and environmental associations. The anticipated findings are expected to deepen our understanding of the diversity and resources of *H. rhamnoides* endophytic fungi, providing a theoretical foundation for exploring cultivable microbial resources, maintaining healthy growth of *H. rhamnoides*, and preventing root diseases. Furthermore, this research will enhance our understanding of how environmental factors influence fungal communities impacting the growth and development of *H. rhamnoides*, thereby offering insights for green ecological protection, vegetation restoration, and the development and utilization of functional flora in the northwest region.

## 2. Materials and Methods

### 2.1. Sample Collection

**Figure 1:**
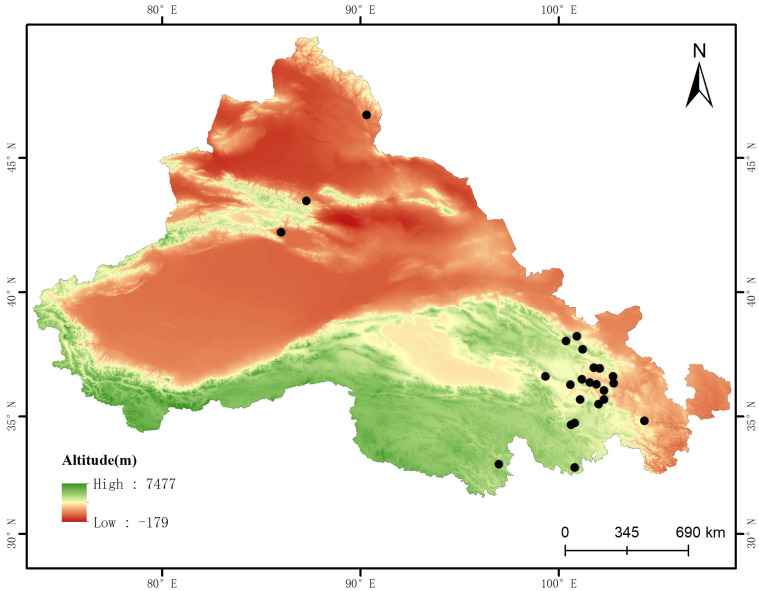
Map of the 25 field sites for fungal communities in the root rhizosphere and endosphere of *H. rhamnoides* in the arid regions of Northwest China.

Root samples of *H. rhamnoides* were collected from 25 distinct locations across the arid regions of Northwest China, encompassing Qinghai, Gansu, and Xinjiang, where the species naturally proliferates. The sites ranged in latitude from 32°87′ N to 46°63′ N and in longitude from 85°59′ E to 104°33′ E. Soil and root samples were collected between late June and the end of August 2023 (Figure 1, Appendix A). Three *H. rhamnoides* plants were randomly obtained from each location, with a minimum distance of greater than 8 m between plants. The *H. rhamnoides* roots were carefully collected using a sterilized shovel, ensuring the collection of fine roots and surrounding soil. The samples were stored in refrigerators for transportation to the laboratory, where impurities such as plant roots and stones were purified from the soil samples, mixed evenly, and sieved through a 2 mm soil sieve, while rhizomes were temporarily stored in an ice box and promptly brought back to the laboratory. The roots were rinsed with running water, followed by three rinses with sterile water, a 30 s soak in 75% ethanol, another rinse, an 8 min soak in 0.2% KMnO_4_, and three final rinses with sterile water to remove non-endophytic fungal interference.

### 2.2. Soil Physicochemical Analyses

The collected soil samples were sieved to remove debris and stored at −20 °C for the determination of their physical and chemical properties. Water content was determined using the drying method (LY/T1213-1999). Soil pH was measured in a 1:2.5 soil-to-water suspension with a pH meter (NY/T 1377-2007) (HSJ-5, INESA Scientific Instrument Co., Ltd., Shanghai, China). Salinity content was determined using the 15% H_2_O_2_ dry weight method (LY/T1251-1999). Total nitrogen (TN) was quantified by an elemental analyzer (LY/T1228-2015) (Elementar EL III, Elementar Analysensysteme GmbH, Langenselbold, Germany). Total phosphorus (TP) was determined with the molybdenum–antimony anti-colorimetric method (HJ632-2011) (T6 UV-Vis, Beijing Purkinje General Instrument Co., Ltd., Beijing, China). Available phosphorus (AP) was measured by the molybdenum–antimony anti-colorimetric method (HJ704-2014) (Multiskan GO 1510, Thermo Fisher Scientific, Vantaa, Finland). Available potassium (AK) was tested with a flame photometer (NY/T889-2004) (Licheng WGH6431, INESA Scientific Instrument Co., Ltd., Shanghai, China). Organic matter (OM) was quantified using the potassium dichromate volumetric method (NY/T1121.6-2006) (T6 UV-Vis, Beijing Purkinje General Instrument Co., Ltd., Beijing, China). Total potassium (TK) was measured with a flame photometer (LY/T1234-2015) (T6 UV-Vis, Beijing Purkinje General Instrument Co., Ltd., Beijing, China). Hydrolyzed nitrogen (HN) was determined using the alkaline diffusion method (LY/T1228-2015) (Elementar EL III, Elementar Analysensysteme GmbH, Langenselbold, Germany).

### 2.3. DNA Extraction, PCR Amplification, and High-Throughput Sequencing

*H. rhamnoides* root samples from various regions were finely ground into powder using liquid nitrogen, with 0.5 g of the resulting powder used for DNA extraction. The extraction followed the protocols outlined in a plant DNA extraction kit, and each sample was processed in triplicate. DNA concentration and purity were evaluated using 1% agarose gel electrophoresis and a nucleic acid analyzer. High-throughput sequencing was subsequently carried out by Beijing Novogene Bioinformatics Technology Co., Ltd. (Beijing, China), employing the universal primer pair ITS1F/ITS2R to amplify the fungal ITS region (ITS1F: 5′-CTTGGTCATTTAGAGGAAGTAA-3′; ITS2R: 5′-GCTGCGTTCTTCATCGATGC-3′). The PCR amplification reaction (20 μL) consisted of 2 μL of 10× Buffer, 2 μL of 2.5 mmol/L dNTPs, 0.8 μL each of the forward and reverse primers, 0.2 μL of rTaq polymerase, 0.2 μL of BSA, 10 ng of template DNA, and ddH_2_O to a total volume of 20 μL. The thermal cycling conditions were as follows: an initial denaturation at 95 °C for 3 min; 35 cycles of 95 °C for 30 s, 55 °C for 30 s, and 72 °C for 45 s; and a final extension at 72 °C for 5 min, with storage at 4 °C.

### 2.4. Statistical Analysis

Environmental factor data for bio1 (annual mean temperature) and bio12 (annual precipitation) were downloaded from the WorldClim website (https://www.worldclim.org/) (accessed on 12 May 2024) at a spatial resolution of 30 arc-seconds to analyze the correlation between these factors and root endophytic fungi. Initially, the original data for each sample were processed by splitting according to the barcode and subsequently removing the barcode and primers. PCR products were then detected, quantified, and used to construct the MiSeq library for Illumina MiSeq PE300 high-throughput sequencing. The R1 and R2 sequence data were then merged using FLASH v1.2.11 software. Quality control was conducted on the merged tags to obtain clean tags, and chimeras were filtered out to yield effective tags for further analysis. The DADA2 algorithm was employed to denoise the effective tags, resulting in the final amplicon sequence variants (ASVs). These ASVs underwent species annotation using the classify-sklearn algorithm in QIIME v2.0, employing a pre-trained naive Bayes classifier for each ASV, and species richness tables were subsequently generated at different classification levels using the software.

Statistical analysis of the sample community composition was performed at various taxonomic levels (kingdom, phylum, class, order, family, genus, and species). Subsequently, dilution curves, relative abundance heat maps, sample distance heat maps [35], and PCoA maps were generated using R4.0.3 software [36]. Functional prediction analysis of fungi was carried out using FUNGuild 1.0, and significant difference analysis of soil physical and chemical properties was performed using SPSS v21.

## 3. Results

### 3.1. Analysis of Soil Physicochemical Properties

The analysis of the physical and chemical properties of *H. rhamnoides* rhizosphere soil across different regions indicates that, except for the PA, GH, and Z3 areas, where the soil pH is mildly acidic, the soil in other regions is predominantly alkaline. Additionally, the northwest region is characterized by arid conditions and low rainfall, resulting in generally low soil water content and high salt levels, as shown in Table 1. Analysis revealed significant correlations between various environmental factors and the pH value, longitude, latitude, and altitude of the sampling points. Specifically, pH values were significantly correlated with TN, HN, TP, and OM across all regions. Longitude showed a significant correlation with AMP, while latitude was significantly correlated with both AMP and AMT. Altitude demonstrated significant correlations with TP, AK, AMP, and AMT. This demonstrates that soil composition varies significantly with changes in geographical environment, leading to variations in physical and chemical properties across different regions.

### 3.2. Quality Analysis of Fungal ITS Sequencing Results

**Figure 2:**
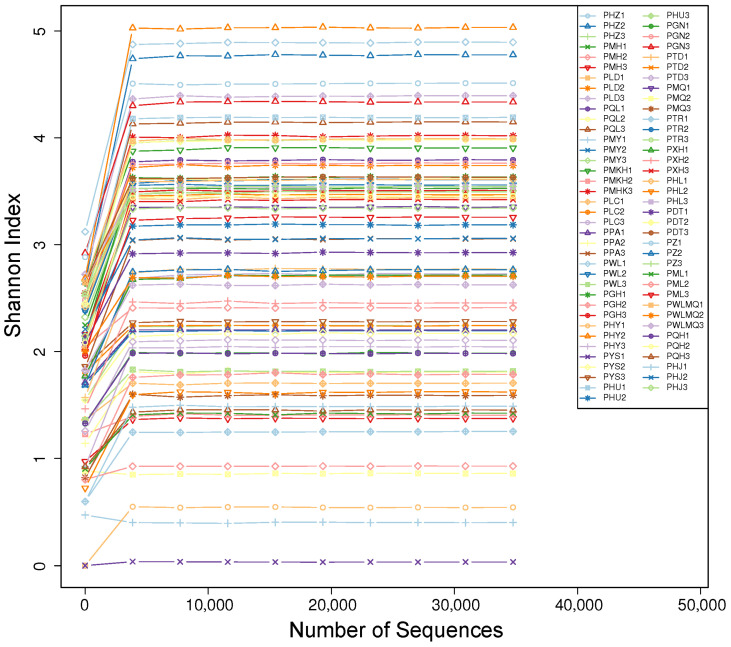
Rarefaction curve analysis for all samples. The horizontal axis represents the sequencing depth, while the vertical axis represents the corresponding alpha diversity index. When the curve levels off, it indicates that the sequencing depth has reached a reasonable point, and additional data will not significantly impact the alpha diversity index.

High-throughput sequencing of 75 *H. rhamnoides* rhizome samples from 25 distinct locations in the arid regions of Northwest China yielded a total of 2,161,934 optimized sequences with an average length of 224 bp. After denoising with the DADA2 method, each unique sequence is referred to as an ASV (amplicon sequence variant), which replaces OTUs (operational taxonomic units) to enhance the accuracy, comprehensiveness, and reproducibility of marker gene data analysis. Significant differences in ASV composition across different regions are shown in Table 2. Constructing a rarefaction curve at 100% similarity is a common method for assessing sample diversity within a group. The rarefaction curve (Figure 2) shows a gradual plateau as the number of extracted sequences increases, indicating that the sample size is sufficient. Further increasing the sequencing data may only capture a few low-abundance species, suggesting that the current sequencing results can realistically represent the fungal community in *H. rhamnoides* rhizosphere samples.

### 3.3. Venn Diagram Analysis

**Figure 3:**
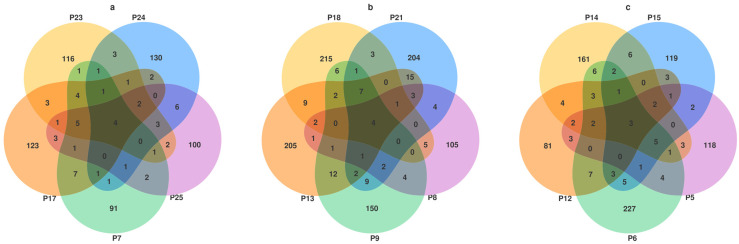
Venn diagrams of endophytic fungus species in *H. rhamnoides* roots at different altitudes at the ASV level. (**a**) Low altitude; (**b**) mid-altitude; (**c**) high altitude. The colors represent different plots, and the numbers represent ASV counts.

Notably, endophytic fungi in *H. rhamnoides* were significantly more abundant in high-altitude areas than in low-altitude areas. The study identified the top ten regions by ASV sequence richness as follows: P19, P6, P13, P21, P18, P4, P11, P1, P20, and P14. The altitude gradient in the study ranged from 1106.3 m to 3665.0 m. Low-altitude areas include P25, P24, P23, P7, and P17, with ASV values of 132, 157, 149, 120, and 159, respectively; high-altitude areas consist of P15, P5, P6, P14, and P12, with ASV values of 146, 154, 190, 202, and 116, respectively. P8, P18, P21, P13, and P9 were chosen based on a 100 m gradient, representing mid-altitude areas, with ASV values of 132, 256, 257, 266, and 200. The Venn diagram constructed based on different altitudes (Figure 3) indicates that P6 and P14, ranking second and tenth in ASV sequence richness, are the two high-altitude regions among the top ten. The total ASV species richness in rhizomes in mid-altitude areas was found to be higher compared to high-altitude and low-altitude areas. The study observed an initial increase followed by a decrease in fungal richness as altitude increased. High-altitude areas exhibited a larger difference in ASV species richness, while low-altitude areas showed a smaller difference.

### 3.4. Alpha Diversity Analysis

**Figure 4:**
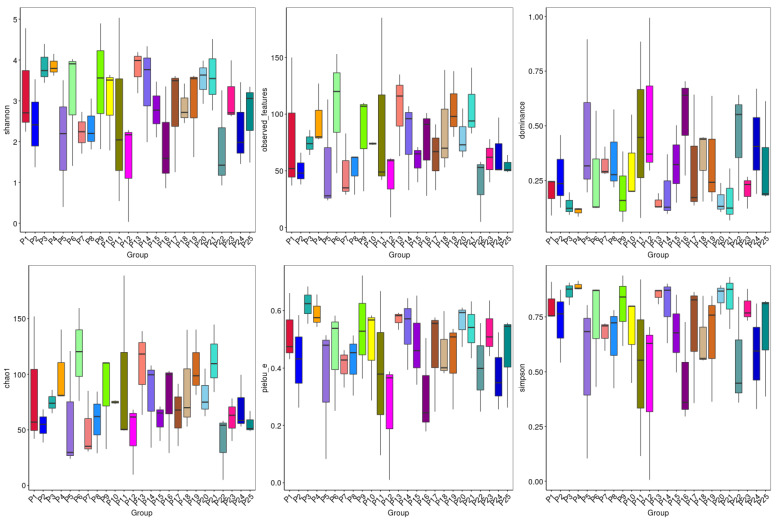
Shannon index, observed features, dominance, Chao1 index, pielou-e, and Simpson index inter-group difference box plot. The horizontal axis of the box plot represents the groups, while the vertical axis represents the corresponding alpha diversity index values. Different colors represent different plots.

Alpha diversity analysis assesses differences in fungus abundance and diversity among groups across various regions. The diversity index analysis of the sequencing results for each sample in this study is depicted in Figure 4 and Table 3. The alpha diversity index was employed for all samples. The Chao1 index indicates fungal community richness, with higher values signifying greater richness. The Shannon and Simpson indices reflect community diversity: higher Shannon index values indicate higher community diversity, whereas higher Simpson index values correspond to lower diversity. Coverage represents the community coverage rate.

**Figure 5:**
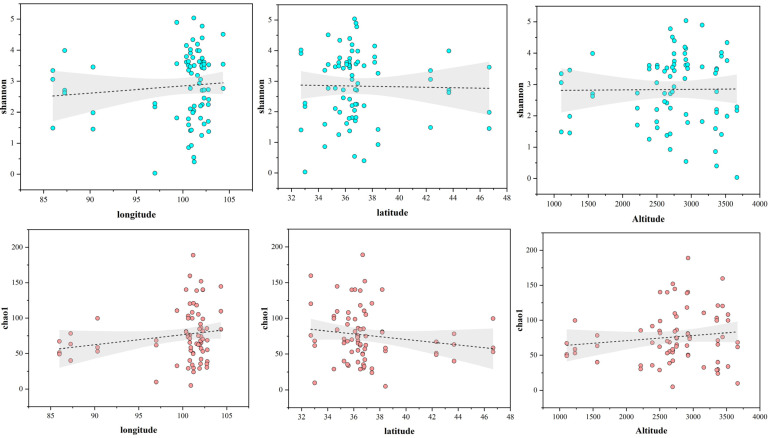
The relationship between endophytic fungus alpha diversity (Shannon and Chao1 indices) and geographical factors (longitude, latitude, and altitude). The different colored circles each represent samples from different sites.

The research findings indicate that the sequencing depth of each sample is satisfactory, with a coverage index exceeding 99%, indicating sufficient detection for community diversity analysis. With increasing altitude, the Chao1 index initially rises and then declines, indicating a moderate increase in microbial abundance. Similarly, the Shannon index initially increases and then decreases with altitude, while the Simpson index exhibits an opposite trend. In terms of pH variation, the soil pH ranges between 6.46 and 8.72 (Table 1). The Chao1 index initially decreases and then rises, plummeting at a pH of 8.05 and sharply increasing at 8.28 (Table 3). Concurrently, the Shannon index also rises. Fungal diversity within *H. rhamnoides* rhizomes across 25 regions varies, with HL exhibiting the highest richness and ML the lowest; QL shows the highest community diversity and ML the lowest. Regional analysis reveals generally high fungal community diversity and richness across samples. Overall, fungal species diversity tends to increase and then decrease with rising altitude (Figure 5). General linear regression is commonly employed to explore the relationship between fungal community alpha diversity and geographical variables such as longitude, latitude, and altitude. The results indicate that the Shannon and Chao1 indices of fungi in *H. rhamnoides* roots do not exhibit significant correlations with longitude, latitude, or altitude.

### 3.5. Beta Diversity Analysis

**Figure 6:**
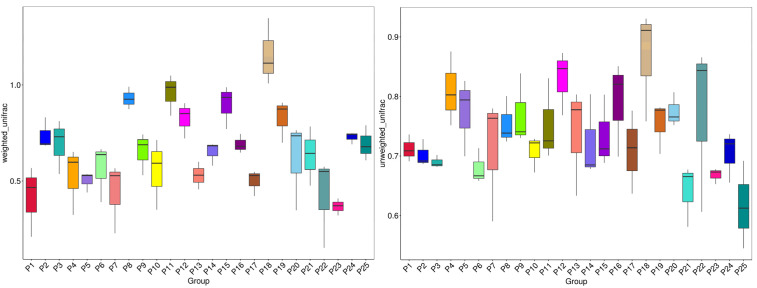
Weighted and unweighted Unifrac distance box plots. Beta diversity analysis reflects the composition of biological communities between different samples. Different colors represent different plots.

**Figure 7:**
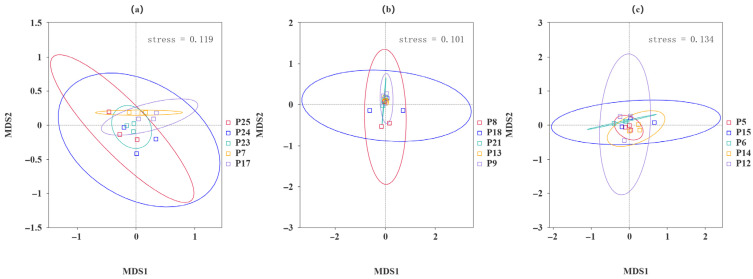
NMDS distribution of fungi in rhizomes of *H. rhamnoides* at different altitudes. (**a**) Low altitude; (**b**) mid-altitude; (**c**) high altitude. NMDS analysis represents samples as points in a multidimensional space, where the degree of difference between samples is reflected by the distance between points. This analysis illustrates both inter-group and intra-group variations among the samples.

Beta diversity analysis of microbial community composition across samples is a crucial concept in ecology. It compares changes in species diversity among ecological communities. This study utilizes box plots to visualize unweighted Unifrac (Figure 6) [37,38], visually representing the distribution of beta diversity across various communities. The NMDS method is employed to analyze the beta diversity distribution in *H. rhamnoides* at varying altitudes, specifically focusing on fungi (Figure 7). By observing the distribution of different points in the plot, one can identify the aggregation patterns and diversity differences of fungi at specific altitudes.

**Figure 8:**
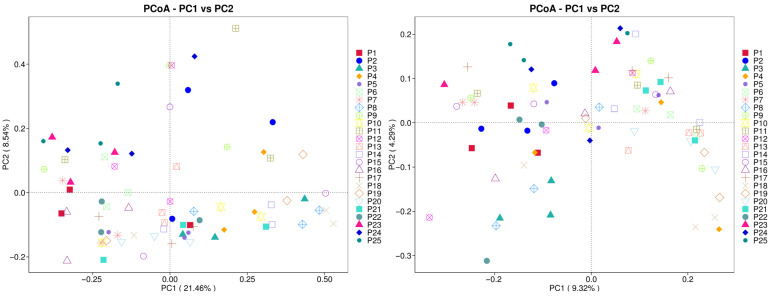
Weighted Unifrac distance 2D PCoA diagrams. The horizontal axis represents one principal component, while the vertical axis represents another principal component. The percentage indicates the contribution of each principal component to the variation among samples. Each point in the plot represents a sample, with samples from the same group denoted by the same color.

The results depicted in Figure 8 illustrate a significant dispersion of samples from high and low altitudes, indicating notable differences and a lack of similarity in the fungal composition of the rhizomes. Conversely, samples from lower-altitude regions exhibit closer clustering, suggesting a relatively similar fungal composition in their roots.

Principal coordinate analysis (PCoA) was performed on *H. rhamnoides* samples from various geographical locations to extract key elements and structures from multidimensional data using eigenvalues and eigenvectors. The analysis utilized both weighted Unifrac and unweighted Unifrac distances, with the principal coordinate combinations yielding the highest contribution rates selected for visualization. The results indicate distinct separations in compositions across different regions. The weighted Unifrac distance PCoA analysis accounted for 30% of the variation, with PC1 explaining 21.46% and PC2 explaining 8.54%. Similarly, the unweighted Unifrac distance PCoA analysis explained 30% of the variation, with PC1 explaining 9.32% and PC2 explaining 4.29%. The proximity of samples P23, P24, and P25 in low-altitude areas suggests a similar species composition structure among them.

### 3.6. Species Composition Analysis

Species with a relative abundance of >1% were considered the dominant fungal groups in this study. The classification of each ASV was performed using the QIIME2 classify-sklearn algorithm with a pre-trained naive Bayes tool for species annotation.

**Figure 9:**
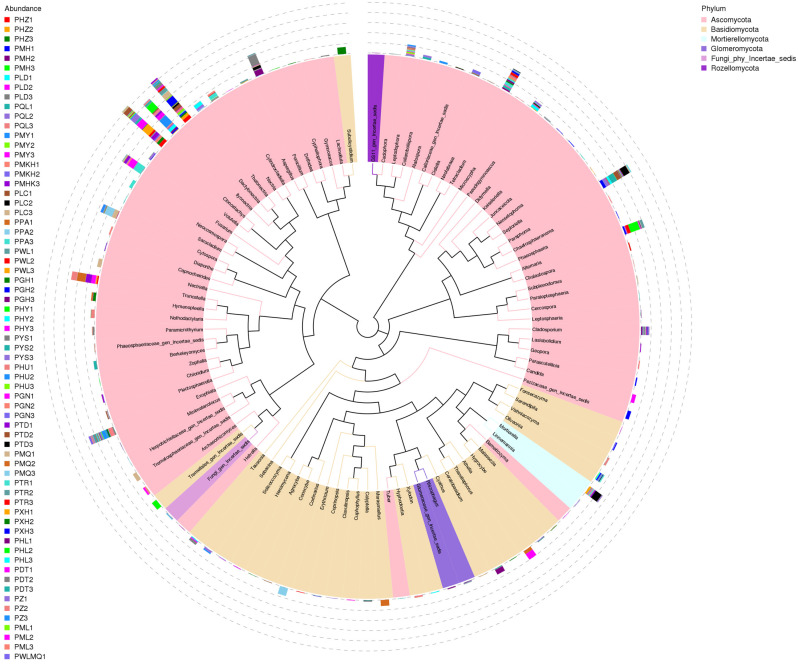
Representative sequences of the top 100 genera. (The colors of branches and sectors indicate their corresponding phylum, while the stacked column chart outside the sector ring represents the abundance distribution information of the genus in different samples).

The number of fungi identified in the rhizomes of *H. rhamnoides* varied across different altitudes. The dominant fungal groups across different regions were primarily Ascomycota, Basidiomycota, Mortierellomycota, and Glomeromycota (Figure 9). The relative abundance of fungal in various plots exceeded 90%. At the genus classification level, representative sequences of the top 100 genera were obtained through sequence alignment, identifying 89 dominant fungal genera, including *Dactylonectria*, *Hymenopleella*, *Truncatella,* and *Mortierella*. These findings suggest a considerable overlap in fungal genera present in *H. rhamnoides* across different regions. The rhizomes exhibited a diverse fungal community structure, with variations in dominant fungal genera observed at different taxonomic levels. Notably, the distribution of each dominant fungal in different sample types showed distinct differences, although common dominant flora such as Penicillium and Paraphoma were identified, with P12 representing the lowest altitude and P25 the highest altitude.

**Figure 10:**
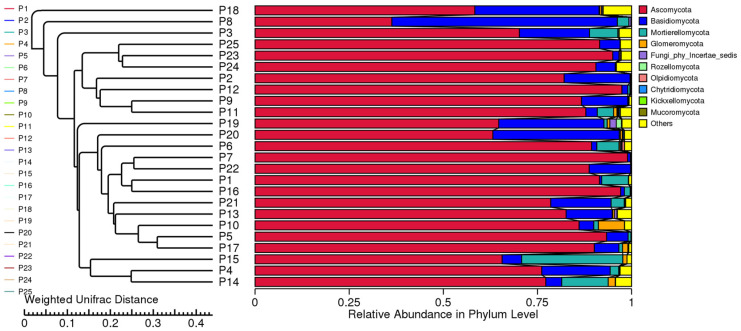
UPGMA clustering tree based on weighted Unifrac distance. On the left is the UPGMA clustering tree structure, and on the right is the relative abundance distribution of species at the phylum level for each sample.

Cluster analysis was conducted on samples from 24 regions to explore similarities. A UPGMA cluster analysis using the weighted Unifrac distance matrix was performed, integrating clustering results with species abundance at the phylum level (Figure 10). The top 10 fungal groups were clustered based on relative abundance at the phylum level, revealing that *H. rhamnoides* samples with similar altitudes cluster together. Dominant phyla identified were *Ascomycota*, *Basidiomycota*, *Mortierellomycota*, and *Glomeromycota*. Variations in altitude were found to influence fungal composition, with rhizome fungi at lower altitudes showing higher structural similarity compared to those at higher altitudes, which consistent with the results of NMDS.

### 3.7. Origin Difference Analysis

**Figure 11:**
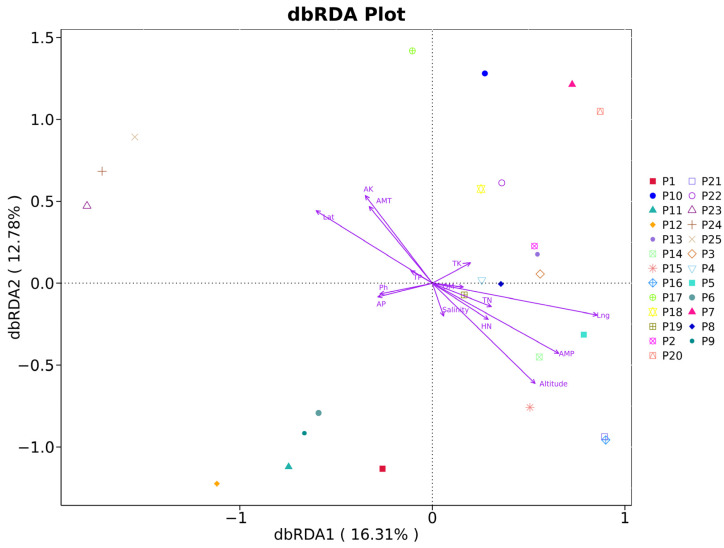
Environmental factors of rhizosphere soil of *H. rhamnoide* and db-RDA analysis. The axes represent major variation components, with arrows indicating the direction and strength of environmental variables. Points represent samples or species, and the proximity to an arrow suggests a stronger association with that variable. Longer arrows indicate a greater influence of the environmental variable.

RDA analysis was conducted on the distribution data and environmental data of *H. rhamnoides* endophyte communities at the genus level across varying altitudes. The results presented in Figure 11 demonstrate that the first and second axes of the RDA explained a cumulative 29.09% of the variance, indicating the influence of soil environment on the fungal communities within the rhizomes. A permutation test (the Mantel test) revealed that soil environmental factors such as AP (r^2^ = 0.598, *p* = 0.027), AK (r^2^ = 0.098, *p* = 0.303), and TN (r^2^ = 0.082, *p* = 0.346) significantly shape the *H. rhamnoide* community, identifying them as key differentiating factors.

**Figure 12:**
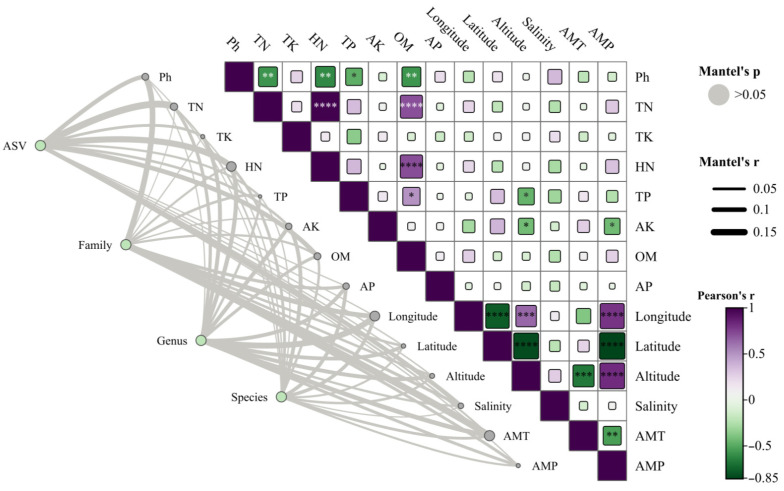
Mantel test correlation heat map of fungi (family, genus, species, and ASV) and environmental factors (AMT, AMP, salinity, altitude, latitude, longitude, AP, OM, AK, TP, HN, TK, TN, and pH) in roots. The colors in the heat map represent the strength of the correlation. The significance levels are as follows: *p* < 0.05, one asterisk (*); *p* < 0.01, two asterisks (**); *p* < 0.001, three asterisks (***); *p* < 0.0001, four asterisks (****).

**Figure 13:**
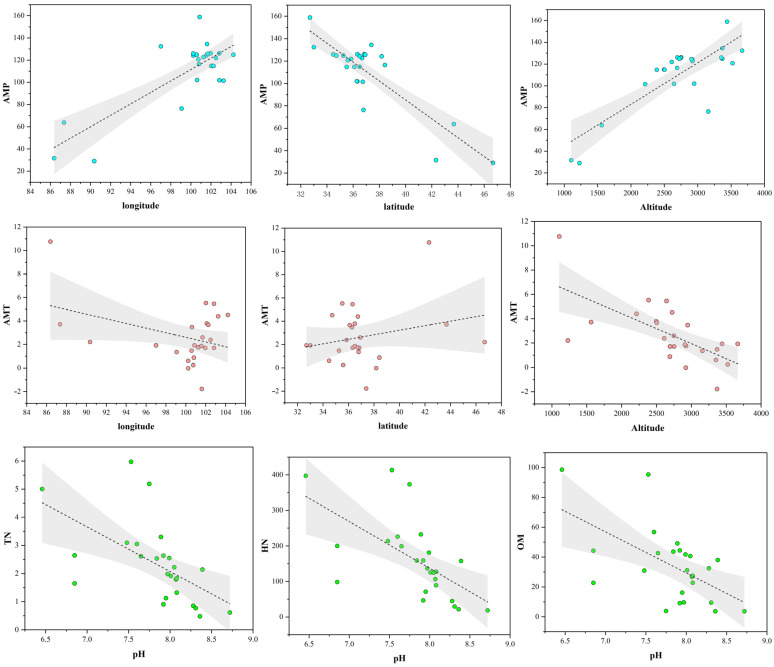
The relationship between soil physicochemical (TN, HN, OM, and pH) and geographical factors (AMT, AMP, altitude, latitude, and longitude). The different colored circles each represent samples from different sites.

The correlation between 14 ecological factors, including longitude, latitude, altitude, AMT (annual mean temperature), AMP (annual mean precipitation), pH, salinity, TN (total nitrogen), TK (total potassium), TP (total phosphorus), AP (available phosphorus), OM (organic matter), AK (available potassium), and HN (hydrolyzable nitrogen), and root-associated fungi of *H. rhamnoides* classified by family, genus, and species, along with their ASV, was examined. Mantel correlation analysis revealed that environmental factors have a relatively minor impact on the root fungi of *H. rhamnoides* (Figure 12), suggesting a high degree of stability in the fungal community within the roots at a large scale. The Mantel test demonstrated a significant correlation between the physical and chemical properties of *H. rhamnoides* soil and variables such as AMT and AMP with altitude, longitude, and latitude. The results from the Pearson analysis can be seen in Figure 13. Specifically, AMP was significantly correlated with longitude (r^2^ = 0.62, *p* < 0.001), latitude (r^2^ = 0.99, *p* < 0.001), and altitude (r^2^ = 0.99, *p* < 0.001). Additionally, AMT was significantly associated with altitude (r^2^ = 0.44, *p* < 0.001). Soil pH showed significant correlations with TN (r^2^ = 0.95, *p* = 0.003), HN (r^2^ = 0.35, *p* = 0.001), and OM (r^2^ = 0.30, *p* = 0.003).

### 3.8. Fungal Community Function Prediction

**Figure 14:**
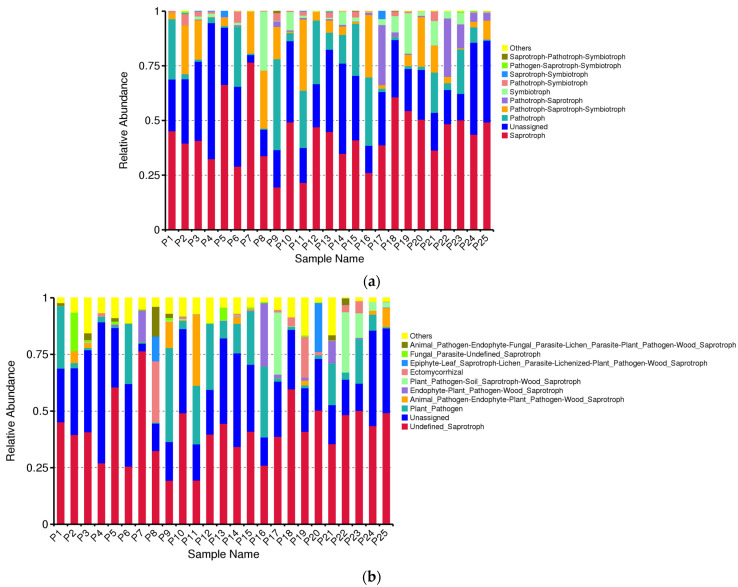
Relative abundance plot. The horizontal axis represents the sample names; the vertical axis indicates the relative abundance. “Others” represents the sum of relative abundances for all functional information not included among the ten features shown in the figure. (**a**) Relative abundance of different trophic types; (**b**) relative abundance bar diagram of ecological function groups.

**Figure 15:**
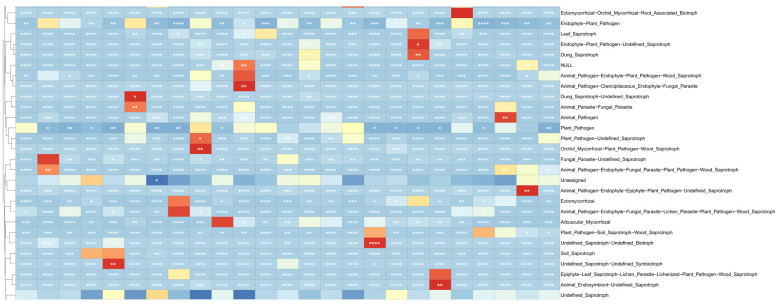
Functional annotations and their abundance information. The heat map displays the correlation between distance matrices, with each cell representing the correlation coefficient between two matrices. The colors indicate the strength and direction of the correlation. The significance levels are as follows: *p* < 0.05, one asterisk (*); *p* < 0.01, two asterisks (**); *p* < 0.001, three asterisks (***); *p* < 0.0001, four asterisks (****).

FUNGuild is a program designed to predict the ecological functions of fungi by analyzing marker gene sequencing profiles. This method offers a cost-effective and reliable alternative to metagenomic research for predicting functional potential. FUNGuild identified nine trophic types, including Saprotroph and Pathotroph–Saprotroph–Symbiotroph. Additionally, 53 ecological co-located groups were predicted across the samples, with the top ten relative abundances including Undefined_Saprotroph and Animal_Pathogen–Endophyte–Plant_Pathogen–Wood_Saprotroph (Figure 14). Notably, the results revealed a higher proportion of plant pathogenic fungi in *H. rhamnoides* from different regions. These asymptomatic plants may harbor pathogenic fungi that remain dormant or act as opportunistic pathogens. Further exploration is needed to unveil the potential physiological functions of these pathogenic fungi. The presence of undefined fungal groups and multiple ecological functional groups for certain fungi may be attributed to limitations in the reference species within the FUNGuild database, highlighting the need to enhance the accuracy of fungal community function prediction. Fungal community function prediction suggests that Saprotroph and Pathotroph–Saprotroph–Symbiotroph are the predominant groups of endophytic fungi in the fine roots of *H. rhamnoides.* The Animal_Pathogen–Endophyte–Plant_Pathogen–Wood_Saprotroph functional group exhibits significant abundances across different altitude conditions and sample variations. Furthermore, a heat map was generated using the top 35 abundant functions and their abundance information from the database (Figure 15), enabling clustering based on functional differences.

### 3.9. Fungal Molecular Network

**Figure 16:**
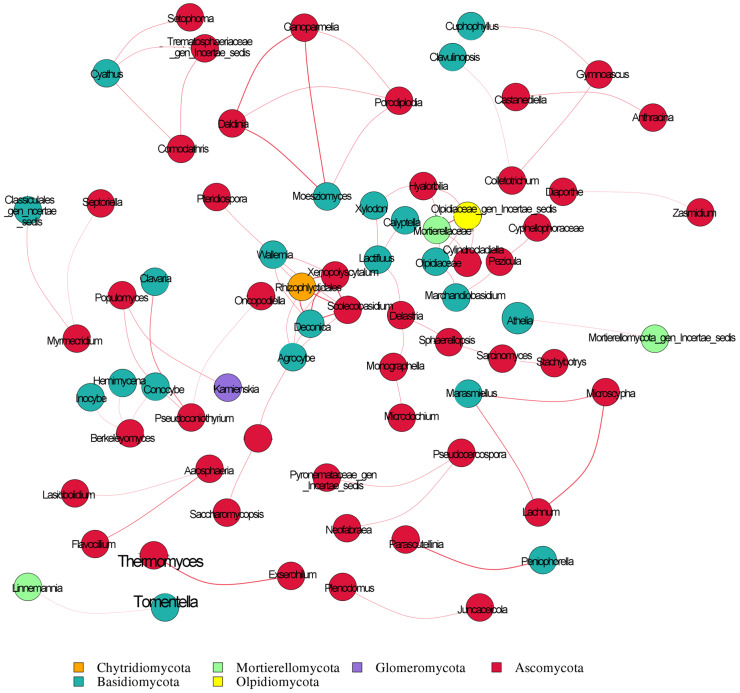
Molecular network diagram of intra-root fungi. The size of each genus represents its average relative abundance. Nodes of the same color represent the same phylum, and the thickness of the edges between nodes is proportional to the absolute value of the species interaction correlation coefficient. Red edges represent positive correlations between genera.

By visualizing the interactions between microorganisms, ecological network analysis can simplify the complex structure of microbial communities. Variations in the types or quantities of fungal microorganisms in the fine roots of *H. rhamnoides* across different regions have led to corresponding changes in network size and complexity. This study compared the topological properties of fungal biocommunities within *H. rhamnoides* from various regions. The network connectivity distribution of fungal communities in the roots of *H. rhamnoides* in 25 regions conforms to the power law model. The clustering coefficient, average distance, and modularity of the microbial domain network differ significantly from those of a corresponding random network, indicating that the interconnections within the microbial network have significant deterministic properties, represented by modular structures (Figure 16). Intra- and inter-module connectivity define topological roles, categorized into module hubs, network hubs, peripheral nodes, and connector nodes. Network hubs and module hubs are highly connected within their respective modules, making them key players in the entire network. By calculating the Spearman correlation index for all samples, the correlation analysis between fungal species in rhizomes from 25 regions was visualized. The analysis identified six genera: *Chytridiomycota*, *Mortierellomycota*, *Glomeromycota*, *Ascomycota*, *Basidiomycota*, and *Olpidiomycota*.

## 4. Discussion

The growth and development of plants are intricately linked to their environment, with soil serving as the primary source of nutrients and root secretions playing a role in altering the soil’s physical and chemical properties. Root microorganisms, as essential components of the plant ecosystem, play a crucial role in the healthy growth of cultivated plants [39]. Numerous studies have demonstrated that root-associated microorganisms significantly impact the soil’s physical and chemical characteristics, and contribute to the creation of a unique soil environment [40,41,42]. Factors influencing root fungal structure vary across habitats, depending on plant type and growth conditions, underscoring the significant role of ‘plant–soil–microbe’ interactions in plant growth [15]. This study aimed to examine the community structure of fungi in the roots of *H. rhamnoides* from various locations, assess their geographical distribution patterns, and investigate their relationship with ecological and environmental factors. The research revealed significant variations in fungal diversity among *H. rhamnoides* roots at different sites, with mid-altitude regions showing higher diversity. This suggests that environmental factors across altitudes play a crucial role in shaping fungal communities within plant roots. Previous studies have indicated that root microbial diversity tends to be lower in high-latitude regions due to harsh climatic conditions like low temperatures, short growing seasons, and limited soil nutrients [43,44]. Conversely, microbial diversity is typically higher in roots from low-latitude regions, where warmer and more humid climates and active soil nutrient cycles create a favorable environment for microorganisms [45]. The alpha and beta diversity indices of fungi in *H. rhamnoides* rhizomes varied across 25 regions, showing differences in richness and community diversity. The highest fungal richness was observed in HL, while the lowest was in ML. Similarly, the highest fungal community diversity was in QL and the lowest in ML. Analysis of different regions indicated generally high fungal community diversity and richness in the samples. Overall, an increase in altitude was associated with an initial rise and subsequent decline in fungal species diversity, supporting the conclusion that fungal community diversity is highest in mid-altitude areas. NMDS and PCoA results revealed minimal differences in fungal structure in roots at mid-altitude areas, with greater dispersion in high- and low-altitude areas but similar species composition structures. Various environmental factors such as climate and soil play crucial roles in plant growth and development [46,47]. Factors such as humidity, temperature, salt content, and soil nutrient levels (e.g., longitude, latitude, altitude, AMT, AMP, pH, salinity, TN, TK, TP, AP, OM, AK, and HN) can significantly impact microorganisms. Research indicates that pH significantly influences microbial diversity and community composition, with neutral soils typically having a more pronounced effect compared to acidic or alkaline soils, as soil pH directly affects the physiological state and ecological niche of fungi, while indirectly influencing fungal communities by regulating the bioavailability of soil nutrients [48,49,50]. This study analyzed soil pH values ranging from 6.24 to 8.42. The results showed that the Chao1 index initially decreased and then increased when pH levels ranged from 6.46 to 8.01. A significant drop in the Chao1 index was observed at a pH of 8.05, followed by a sharp increase until reaching 8.28. Similarly, the Shannon index exhibited a decline followed by a sharp increase around the pH value of 8.05, while the Simpson index displayed an initial sharp increase followed by a decrease. These fluctuations were attributed to the proliferation of alkali-resistant fungi in alkaline soil. Previous studies have indicated that alkaline soil supports a higher abundance and diversity of saprotrophic fungi compared to neutral soil, as well as a richer and more diverse fungal community in the roots of *H. rhamnoides* [51,52]. Several studies have indicated that latitude, longitude, and annual precipitation play significant roles in fungal richness. Utilizing RDA and Mantel test analyses, this study identified longitude, AK, and HN as the primary factors influencing the variations in fungal communities within the rhizomes of *H. rhamnoides.* These findings provide additional evidence that geographical environmental factors drive the diversity of fungi in the roots of *H. rhamnoides*. Examination of fungi within the roots revealed that the dominant fungal groups belonged to four phyla: Ascomycota, Basidiomycota, Mortierellomycota, and Glomeromycota, with their combined relative abundance exceeding 90% in various plots. The taxonomic composition and relative abundance of dominant fungal groups varied across different regions, further supporting the notion that the geographical environment influences the richness of the fungal community. The findings of this study indicate that the fungal communities within *H. rhamnoides* roots exhibit regional variations influenced by environmental and geographical factors. The enrichment and depletion of certain fungi in *H. rhamnoides* suggest that plants can actively select fungal colonization. The diversity of fungi in plant rhizomes may not be solely induced by plant actions alone; interactions among microorganisms also play a significant role [53]. The 14 environmental factors investigated in this study comprehensively affect rhizosphere fungal communities and accurately reflect the relationship between plants and their environments.

## 5. Conclusions

A comprehensive study on the diversity of fungal communities in *H. rhamnoides* rhizomes across 25 regions revealed substantial fungal community and functional diversity in each region. Notably, both fungal community and functional diversity were higher in mid-altitude areas compared to low and high altitudes. Additionally, the intra-root similarity of fungal samples from low-altitude areas was higher than those from mid-altitude and high-altitude areas. The relative abundance of dominant fungal genera varied between different sampling points, while the community structure of samples from the same area exhibited high similarity. Among various ecological factors, including longitude, latitude, altitude, AMT, AMP, pH, salinity, TN, TK, TP, AP, OM, AK, and HN, it was found that environmental factors such as longitude, altitude, and soil AK play crucial roles in shaping the *H. rhamnoides* rhizome community. The species diversity and primary ecological functions of fungi in *H. rhamnoides* roots were found to vary based on their geographic origin, suggesting that the accumulation or depletion of various substances during growth leads to the formation of specific fungal communities in each region. These findings elucidate the diversity characteristics of fungi in *H. rhamnoides* rhizomes across different regions and provide valuable insights for the sustainable development and utilization of microorganisms in the rhizomes of *H. rhamnoides* in the northwest region.

## Figures and Tables

**Table 1:** Physicochemical property information of rhizosphere soil at sampling sites.

Sample	pH	Moisture	Salinity	TNg/kg	TKg/kg	HNmg/kg	TPg/kg	AKmg/kg	OMg/kg	APmg/kg
P1	7.600	14.519	3.270	3.050	15.489	225.554	0.753	39.065	56.772	6.735
P2	8.070	11.093	4.327	1.787	19.045	106.049	0.636	67.980	26.704	2.097
P3	7.530	32.745	1.613	5.972	20.600	413.271	0.991	157.386	95.352	2.847
P4	7.990	7.209	2.433	2.550	18.337	181.034	0.635	47.380	41.769	1.204
P5	8.080	18.195	4.180	1.321	19.339	89.088	0.516	21.388	22.782	0.929
P6	7.650	23.879	1.067	2.612	17.951	198.994	0.586	21.164	42.655	3.069
P7	7.920	10.702	4.637	2.634	19.223	158.822	0.721	89.629	44.497	2.832
P8	6.850	17.726	1.457	2.645	13.982	199.854	1.464	19.008	44.269	0.795
P9	7.920	4.222	5.257	0.903	18.325	46.434	0.560	87.477	9.041	3.696
P10	6.850	17.632	0.843	1.649	17.965	98.343	0.591	215.698	22.729	1.254
P11	8.390	16.960	0.910	2.140	16.081	157.388	0.747	85.091	38.031	7.981
P12	8.080	3.721	5.050	1.832	18.272	127.150	0.532	90.198	27.458	6.109
P13	8.010	19.759	0.967	1.913	18.256	124.927	0.615	28.365	31.179	2.090
P14	8.360	7.813	0.883	0.476	18.374	21.179	0.366	18.443	3.652	0.511
P15	8.720	7.616	5.940	0.613	20.679	18.010	0.551	36.399	3.604	0.636
P16	7.750	22.009	1.943	5.187	20.630	373.019	0.571	33.415	3.863	3.306
P17	8.050	7.439	3.607	2.222	17.206	124.072	0.676	139.983	40.595	2.699
P18	8.280	11.779	0.797	0.853	18.201	44.853	0.613	54.288	32.450	17.454
P19	7.970	12.272	1.780	1.981	17.660	136.579	0.679	152.801	9.588	3.972
P20	7.480	12.288	1.750	3.092	19.510	213.332	0.514	66.059	30.930	3.160
P21	6.460	28.396	0.697	5.003	18.552	397.373	1.167	42.630	98.584	3.389
P22	7.840	12.830	1.570	2.536	19.443	158.571	0.923	135.010	43.617	1.182
P23	7.890	7.220	1.480	3.299	19.760	232.105	0.793	153.395	49.156	7.157
P24	8.310	8.249	1.213	0.770	18.392	29.043	1.252	144.198	9.455	5.728
P25	7.950	6.948	1.247	1.124	18.582	70.907	0.608	84.829	16.131	0.365

**Table 2:** Species classification of sampling sites.

Sample	Phylum	Class	Order	Family	Genus	Species	ASV
P1	6	13	25	39	52	53	216
P2	5	13	29	51	65	65	126
P3	6	18	32	58	73	78	197
P4	6	21	41	73	91	84	250
P5	5	17	31	48	57	58	146
P6	8	18	40	62	84	91	271
P7	6	13	28	45	53	54	120
P8	6	17	30	37	54	51	132
P9	6	15	33	57	82	83	200
P10	5	11	24	37	57	62	196
P11	8	17	36	61	86	91	236
P12	7	13	27	37	46	51	116
P13	7	19	39	62	100	101	266
P14	5	15	34	51	67	70	202
P15	4	12	26	44	58	55	154
P16	6	17	37	64	96	88	190
P17	5	12	28	54	68	63	159
P18	7	17	36	56	70	68	256
P19	8	21	45	66	88	93	281
P20	8	19	39	57	70	72	210
P21	8	19	43	74	104	99	257
P22	3	12	26	35	47	47	95
P23	7	13	30	49	53	54	149
P24	6	15	29	46	59	61	157
P25	5	10	26	33	44	47	132

**Table 3:** Alpha diversity index for all samples.

Sample	Chao1	Dominance	Goods_Coverage	Observed_Features	Pielou_e	Shannon	Simpson
P1	232.167	0.1	1	217	0.561	4.357	0.9
P2	134.5	0.096	1	127	0.551	3.85	0.904
P3	199.2	0.07	1	198	0.638	4.868	0.93
P4	266.545	0.051	1	251	0.63	5.021	0.949
P5	156.545	0.253	1	147	0.416	2.997	0.747
P6	280.462	0.111	1	270	0.528	4.262	0.889
P7	125.5	0.191	1	121	0.438	3.031	0.809
P8	149.5	0.121	1	133	0.548	3.866	0.879
P9	206.077	0.093	1	201	0.58	4.438	0.907
P10	199.1	0.158	1	197	0.519	3.953	0.842
P11	242.111	0.16	1	236	0.503	3.968	0.84
P12	123.273	0.187	1	115	0.441	3.018	0.813
P13	272.091	0.065	1	265	0.619	4.984	0.935
P14	204.6	0.077	1	201	0.614	4.696	0.923
P15	153.083	0.11	1	153	0.585	4.247	0.89
P16	200.4	0.197	1	189	0.431	3.257	0.803
P17	161	0.134	1	158	0.534	3.901	0.866
P18	256.5	0.117	1	255	0.548	4.379	0.883
P19	285.833	0.118	1	280	0.536	4.354	0.882
P20	212.273	0.077	1	209	0.592	4.567	0.923
P21	274.4	0.062	1	256	0.614	4.914	0.938
P22	95.667	0.238	1	94	0.432	2.831	0.762
P23	150.545	0.147	1	148	0.522	3.763	0.853
P24	165.067	0.186	1	156	0.473	3.444	0.814
P25	132.5	0.135	1	131	0.544	3.824	0.865

## Data Availability

No new data were created or analyzed in this study.

## Appendix A

**Table A1:** Sampling Plot Information of *H. rhamnoides*.

Sample	Sample Name	AddPess	Soil Type	Sample No.	Latitude (N°)	Longitude (E°)	Altitude /m	AMT	AMP
HZ	P1	Huzhu County, Haidong City	Sand	PHZ1, PHZ2, PHZ3	36.977	102.077	2696.3	1.715	126.085
MH	P2	Minhe County, Haidong City	Loam	PMH1, PMH2, PMH3	36.376	102.782	2644.8	5.456	101.830
LD	P3	Ledu District, Haidong City	Loam	PLD1, PLD2, PLD3	36.978	102.077	2753.5	1.715	126.085
QL	P4	Qilian County, Haibei Tibetan Autonomous Prefecture	Sand	PQL1, PQL2, PQL3	38.076	100.378	2920.8	−0.019	124.205
MY	P5	Menyuan County, Haibei Tibetan Autonomous Prefecture	Clay	PMY1, PMY2, PMY3	37.755	101.220	3369.0	−1.769	134.440
MKH	P6	Make River, Luoguoluo Tibetan Autonomous Prefecture	Clay	PMKH1, PMKH2, PMKH3	32.874	100.820	3441.0	1.925	158.825
LC	P7	Liancheng, Lanzhou City	Loam	PLC1, PLC2, PLC3	36.661	102.736	2214.6	4.394	101.465
PA	P8	Ping’an District, Haidong City	Loam	PPA1, PPA2, PPA3	36.338	101.914	2497.8	3.779	114.900
WL	P9	Wulan County, Haixi Mongolian and Tibetan Autonomous Prefecture	Sand	PWL1, PWL2, PWL3	36.660	99.341	3159.0	1.356	76.410
GH	P10	Gonghe County, Hainan Tibetan Autonomous Prefecture	Clay	PGH1, PGH2, PGH3	36.311	100.595	2947.6	3.467	102.030
HY	P11	Huangyuan County, Xining City	Loam	PHY1, PHY2, PHY3	36.545	101.184	2923.1	1.756	122.610
YS	P12	Yushu Tibetan Autonomous Prefecture	Loam	PYS1, PYS2, PYS3	33.005	96.992	3665.0	1.927	132.345
HU	P13	Huangzhong District, Xining City	Clay	PHU1, PHU2, PHU3	36.400	101.575	2906.8	1.863	124.360
GN	P14	Guinan County, Hainan Tibetan Autonomous Prefecture	Loam	PGN1, PGN2, PGN3	35.707	101.081	3522.1	0.252	120.735
TD	P15	Tongde County, Hainan Tibetan Autonomous Prefecture	Loam	PTD1, PTD2, PTD3	34.736	100.806	3369.0	1.473	124.870
MQ	P16	Maqin County, Guoluo Tibetan Autonomous Prefecture	Loam	PMQ1, PMQ2, PMQ3	34.660	100.622	3353.5	0.615	125.880
TR	P17	Tongren City, Huangnan Tibetan Autonomous Prefecture	Clay	PTR1, PTR2, PTR3	35.518	102.019	2387.9	5.525	114.660
XH	P18	Xunhua Salar Autonomous County, Haidong City	Clay	PXH1, PXH2, PXH3	35.716	102.287	2610.6	2.383	121.885
HL	P19	Haidong Hualong Hui Autonomous County	Clay	PHL1, PHL2, PHL3	36.073	102.280	2503.9	3.667	114.750
DT	P20	Haidong Hualong Hui Autonomous County	Clay	PDT1, PDT2, PDT3	37.002	101.771	2748.7	2.596	125.640
Z	P21	Dingxizhang County, Gansu Province	Clay	PZ1, PZ2, PZ3	34.823	104.330	2725.2	4.510	124.825
ML	P22	Minle County, Gansu Province	Clay	PML1, PML2, PML3	38.265	100.915	2692.5	0.881	116.380
WLMQ	P23	urumqi county of xinjiang	Clay	PWLMQ1, PWLMQ2, PWLMQ3	43.451	87.267	1562.5	3.717	63.720
QH	P24	Qinghe County, Altay Prefecture, Xinjiang Uygur Autonomous Region	Sand	PQH1, PQH2, PQH3	46.532	90.319	1230.0	2.206	29.035
HJ	P25	Hejing County, Bayinguoleng Mongolian Autonomous Prefecture, Xinjiang	Sand	PHJ1, PHJ2, PHJ3	42.282	85.992	1106.3	10.763	31.560
